# Identification of IGF2BP3 for the Expression and Prognosis in Gastrointestinal Cancers

**DOI:** 10.7150/ijms.114411

**Published:** 2025-06-23

**Authors:** Fengfeng Han, Chen He, Chen Qian, Yujie Wang, Mingge Zhou, Ruirui Yang, Zhou Zhang

**Affiliations:** 1 Department of Nuclear Medicine, The Third Affiliated Hospital of Soochow University, Changzhou 213003, Jiangsu, China.; 2 Department of Clinical Laboratory, Xishan People's Hospital of Wuxi City, Wuxi 214105, Jiangsu, China.; 3 Central Laboratory, Xishan People's Hospital of Wuxi City, Wuxi 214105, Jiangsu, China.; 4 Department of Pathophysiology, School of Clinical and Basic Medicine, Shandong First Medical University, Jinan 250000, Shandong, China.

**Keywords:** IGF2BP3, gastrointestinal cancers, diagnosis, prognosis

## Abstract

**Purpose**: This study investigates the expression, diagnostic, and prognostic significance of IGF2BP3 in gastrointestinal cancers, providing new insights for clinical management and patient care.

**Methods**: An integrated approach was used to analyze the expression and mutation of IGF2BP3 across various cancers, utilizing multiple online analytical tools. Data from the TCGA database were examined to identify differentially expressed genes (DEGs) in four types of gastrointestinal cancers and to assess the specific expression of IGF2BP3. Further validation was carried out through western blotting (WB), quantitative real-time PCR (qRT-PCR), and immunohistochemistry (IHC). Protein-protein interaction (PPI) network was also employed to investigate the relationships between IGF2BP3 and its associated genes. Additionally, bioinformatics databases were used to investigate the impact of IGF2BP3 on the diagnosis and prognosis of these cancers.

**Results**: High expression of IGF2BP3 is a notable feature across these four gastrointestinal cancers, as demonstrated in public databases. Confirmation in cancer cells and tissues support this observation. Clinicopathological analysis reveals significant associations between IGF2BP3 expression and cancer stages in gastric cancer, and with cancer grades in liver cancer. The diagnostic accuracy of IGF2BP3 is found to be highest for esophageal cancer. Moreover, elevated IGF2BP3 levels correlate with poorer overall survival (OS) and progression-free survival (PFS) in liver cancer patients.

**Conclusions**: This study underscores the significant expression of IGF2BP3 in gastrointestinal cancers and its potential value in both diagnostic and prognostic assessments. These findings could enhance diagnostic accuracy and support the development of targeted therapeutic strategies.

## 1. Introduction

Gastrointestinal cancers are the most prevalent type of malignancy, constituting roughly 25% of all cancer cases and accounting for about one-third of cancer-related fatalities globally 1. The rising occurrence of these cancers is intricately associated with demographic shifts, such as population expansion, aging populations, and alterations in lifestyle 2. These malignancies primarily encompass esophageal, gastric, liver, and colorectal cancer 3. The early identification of conditions such as esophageal, gastric, and liver cancer is currently inadequate, leading to generally unfavorable patient outcomes 4. Consequently, it is essential to pinpoint innovative diagnostic and therapeutic targets to enhance early detection and treatment, thereby establishing a scientific basis for future treatment protocols.

Some literatures reveal that IGF2BP3 (Insulin-like growth factor 2 mRNA-binding protein 3) is considered an oncogene in multiple tumors, including leukemia 5, glioma 6, oral cancer 7, among others 8. However, the role of IGF2BP3 in gastrointestinal tumors and its potential as a biomarker for gastrointestinal tumors are worth more exploring and summarizing. IGF2BPs form a conserved group of RNA-binding proteins (RBPs) 9, 10. RBPs 11-14 are proteins that bind to RNA, forming ribonucleoprotein complexes. These proteins are predominantly functional in a methylated state, notably in N6-methyladenosine (m6A) 15-17. The research of Oh et al points to the fact that the upregulation of m6A-reading proteins, particularly IGF2BP3, is connected to adverse survival, increased proliferation, and epithelial-mesenchymal transition (EMT) activation across diverse cancers, including gastric and colorectal carcinomas 18. Studies have shown that IGF2BPs are critical in various cellular functions, including cell polarization, migration, morphology, metabolism, proliferation, and differentiation 19. Among them, IGF2BP3, notably abundant in pancreatic cancer tissues and initially named KOC, is particularly overexpressed in various human cancers. Beyond influencing growth and chemoresistance, IGF2BP3 also enhances the invasive capacity of tumor cells in vitro 20-22. Upregulation of this protein in cancers is known to promote tumor growth by increasing IGF2 expression. Additionally, IGF2BP3 aids in tumor cell proliferation by interacting with HNRNPM in the nucleus 23, thereby boosting cyclin protein expression.

The field of bioinformatics, which integrates genomics, information science, mathematics, and biology, is crucial in managing, processing, and interpreting large-scale biological data. It helps uncover the biological significance of such data, facilitating the identification of key tumor biomarkers and laying a foundation for enhanced cancer diagnosis and prognosis 24-26.

In this study, we applied bioinformatics techniques to explore the relationship between IGF2BP3 expression and gastrointestinal cancers, focusing on gene mutations, pathological features, diagnostic potential, and prognostic value. Our findings offer insights that could guide future research into the role of IGF2BP3 in these cancers.

## 2. Materials and methods

### 2.1 Expression and mutation analysis of IGF2BP3 in pan-cancer

The expression patterns of IGF2BP3 across pan-cancer tissues were assessed using the University of Alabama at Birmingham Cancer Data Analysis Portal (UALCAN). Afterwards, extensive genomic data, representing various cancer types, was analyzed using the cBioPortal platform. This data included mutation frequency, variant categorization, copy number modifications, and mutation localization.

### 2.2 Gene mutations and related genes analysis of IGF2BP3 in gastrointestinal cancers

Initially, the cBioPortal platform was used to examine the origins and patterns of IGF2BP3 mutations in gastrointestinal cancers. Further analysis through the UALCAN database identified significant correlations between IGF2BP3 and its core regulatory genes across prevalent gastrointestinal cancers types, including the quantification of their respective correlation coefficients.

### 2.3 Identification of DEGs in gastrointestinal cancers

To show the gene differential expression landscape among four gastrointestinal cancers types, volcano plots were made using datasets from The Cancer Genome Atlas (TCGA) 4. Statistical analysis was then performed to identify significant differences, which were shown in box plots, after the IGF2BP3 expression levels were quantified using TCGA data.

### 2.4 Protein-protein interaction (PPI) networks in gastrointestinal cancers

The online database STRING was used to analyze the functional interactions between IGF2BP3 and its correlated genes including positive and negative correlated genes in gastrointestinal cancers.

### 2.5 Survival analysis and diagnostic efficacy of IGF2BP3 in gastrointestinal cancers

Using TCGA cohort data, this study evaluated the prognostic significance of IGF2BP3. Subsequent ROC curve analysis quantified diagnostic performance through systematic comparison of AUC values and threshold optimization, identifying the statistically optimal discrimination cutoff for clinical application.

### 2.6 Cell lines and cell culture

The Cell Bank of the Chinese Academy of Sciences (China) was used to procure the following human cancer cell lines: TE-1 (esophageal cancer), HGC-27 (gastric cancer), HepG2 (liver cancer), and HT-29 (colorectal cancer). The following human cell lines were obtained from IMMOCELL: HET-1A (esophageal epithelium), GES-1 (gastric epithelium), LX-2 (hepatocyte), and NCM460 (colorectal epithelium). All cells, except for HET-1A cells, were cultured in RPMI-1640 with 10% FBS, while HET-1A cells were grown in a distinct medium. All cells were kept at 37°C in 5% CO_2_.

### 2.7 Quantitative real-time PCR (qRT-PCR)

The Trizol reagent (Ambion) was employed for the extraction of total RNA. The application of Vazyme's HiScript® II Q Select RT SuperMix facilitated the reverse transcription process for qRT-PCR. To quantify mRNA levels, qRT-PCR was conducted utilizing the SYBR Green Master Mix. The primer sequences of IGF2BP3 were as follows: Forward, 5'-GGCAAAACGGTGAATGAACT-3' and Reverse, 5'-GTCCACTTTGCAGAGCCTTC-3'. Using β-actin as an internal reference gene and the primer sequence were as follows: Forward, 5'-CCCTGGAGAAGAGCTACGAG -3'and Reverse, 5'-CGTACAGGTCTTTGCGGATG -3'. The 2^-ΔΔCt^ method was employed to assess the levels of IGF2BP3. There were three iterations of the experiments.

### 2.8 Western blotting (WB)

The whole cell protein was isolated for Western blotting by transferring it to PVDF membranes (Millipore, Billerica, MA, USA) after being separated by 12% SDS-PAGE in RIPA lysis buffer (Invitrogen, Carlsbad, CA, USA). Priming antibodies: rabbit anti-IGF2BP3 (diluted 1:5000; Proteintech, China) and mouse anti-β-actin (dilution 1:20000, Affinity, China) were applied to the membranes after blocking them with 5% non-fat milk and incubating them overnight at 4°C. Proteintech of China diluted goat anti-rabbit/mouse secondary antibodies 1:2000 into the membranes and left them to incubate at 37°C for 60 minutes. Millipore, Germany's ECL reagents were used to identify signals after incubation. As an internal control, β-actin was utilized. There was at least one triplicate of each experiment conducted separately.

### 2.9 Immunohistochemistry (IHC)

The IHC was carried out using a kit from Boster Biological Technology, China, in accordance with the instructions provided by the manufacturer. Thin slices measuring 5 μm were cut from tumor tissues that had been fixed in paraffin. Dewaxing and rehydration were followed by the application of 3% H_2_O_2_ to inactivate endogenous peroxidase. Primary antibodies against IGF2BP3 (diluted 1:200; Proteintech, China) were incubated overnight at 4°C after blocking the slices with 5% BSA for 1 hour. Slices were stained with 3,3-diaminobenzidine substrate (DAB) after incubation with the secondary antibody such that IGF2BP3 expression could be observed under a microscope.

### 2.10 Statistics analysis

Data analysis was conducted using R software (version 4.02) and presented as mean ± SD. Statistical differences were assessed using GraphPad Prism software 8.0.2 (USA). Differential gene expression analysis (|Log2FC| > 1, adjusted *P*-value (FDR) < 0.05) was performed by comparing tumor tissues with normal controls using the limma R package. Gene expression data were log2-transformed, and correlations were calculated using Spearman's rank correlation coefficient. The diagnostic potential of IGF2BP3 in gastrointestinal cancers was evaluated through receiver operating characteristic (ROC) curves. Survival analysis was conducted using Kaplan-Meier survival curves to assess the prognostic value of IGF2BP3 expression in these cancers. Statistical significance was determined using either an independent t-test for two groups or one-way analysis of variance (ANOVA), with a *P*-value of <0.05 considered significant. *P<0.05, **P<0.01, ***P<0.001.

## 3. Results

### 3.1 Clinical parameters of patients

Clinical datasets for four cancer types were systematically retrieved from TCGA. The cohort included 184 validated esophageal cancer cases, 403 qualified gastric cancer samples, 349 confirmed liver cancer specimens, and 404 validated colorectal cancer records that met the inclusion criteria (Table 1).

### 3.2 Expression and mutation of IGF2BP3 in pan-cancer

**Figure 1:**
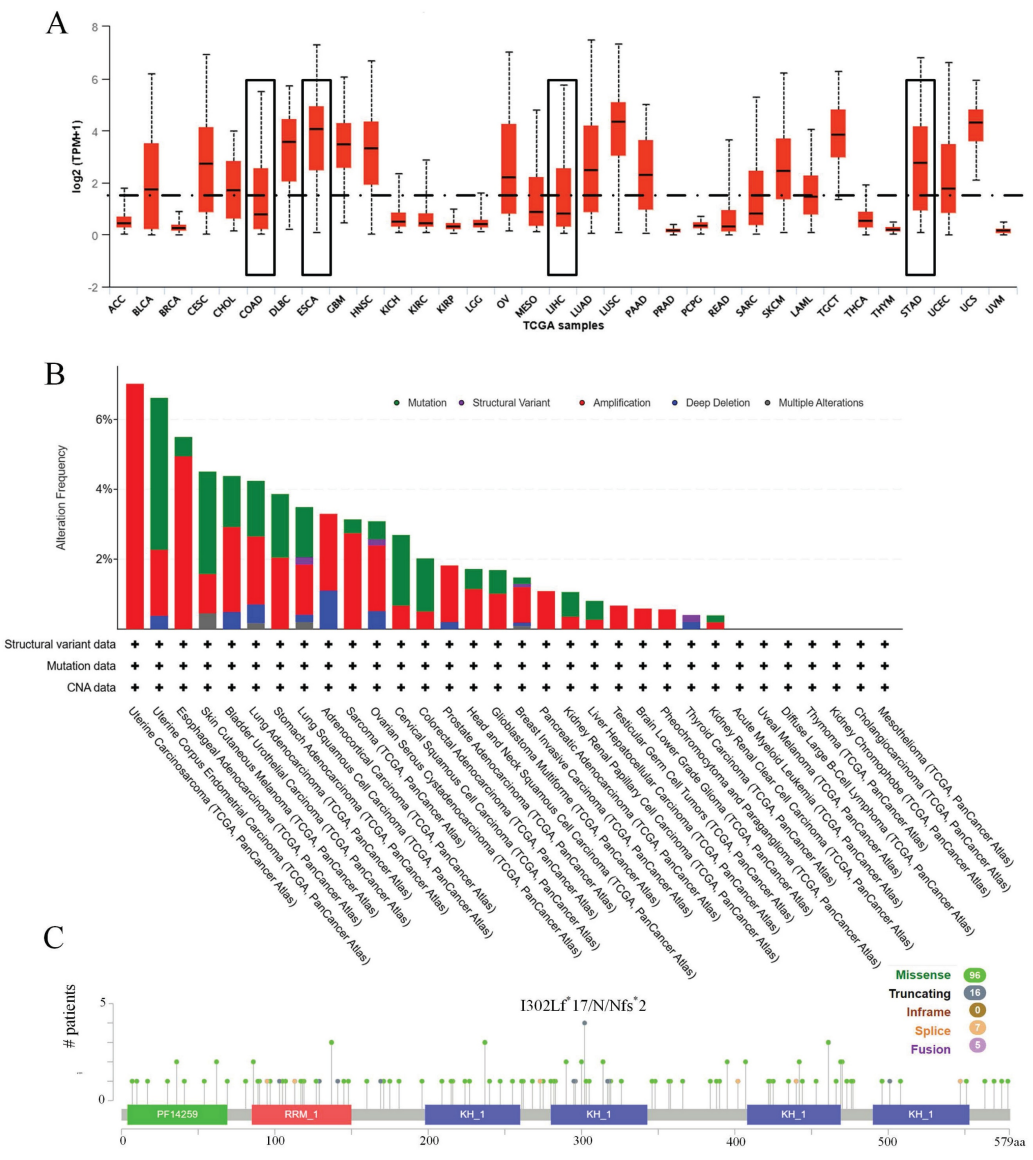
Expression and mutation of IGF2BP3 in pan-cancer. (A) Expression of IGF2BP3 in pan-cancer. (B) Frequency and types of alterations in IGF2BP3 across pan-cancer. (C) Gene mutation sites of IGF2BP3 in pan-cancer.

Initially, the expression profile of IGF2BP3 across various cancers was examined. Figure 1A demonstrates that IGF2BP3 expression is higher in esophageal cancer and gastric cancer compared to the median expression across pan-cancer. Additionally, the mutation frequency, types of mutations, and copy number variations of IGF2BP3 were analyzed. Figure 1B displays the frequency and types of alterations in IGF2BP3 across pan-cancer. The main alteration in esophageal cancer is amplification, with a frequency of 4.95%. In liver cancer, the main alteration is mutation, with a frequency of 0.54%. In gastric cancer, amplification is the primary alteration, with a frequency of 2.05%. In colorectal cancer, the primary alteration is mutation, with a frequency of 1.52%. Figure 1C shows multiple mutation sites of IGF2BP3 across pan-cancer.

### 3.3 Expression and gene mutations of IGF2BP3 in gastrointestinal cancers

**Figure 2:**
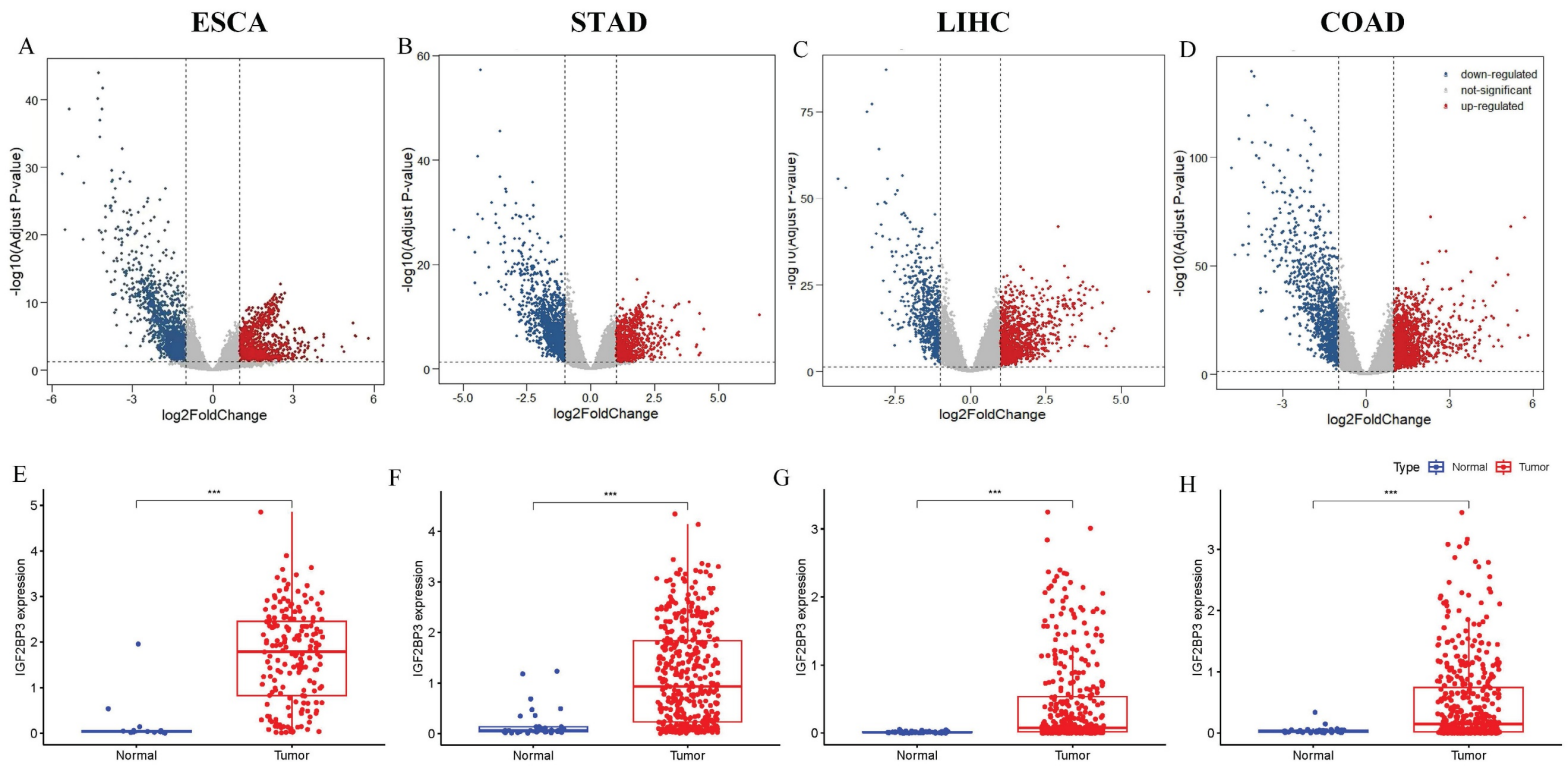
Expression of IGF2BP3 in gastrointestinal cancers. (A-D) Volcano plots of gastrointestinal cancers. (E-H) Box-plots of IGF2BP3 in gastrointestinal cancers.

Next, the expression profile of IGF2BP3 in gastrointestinal cancers was explored. DEGs in gastrointestinal cancers were identified using R programming. Volcano plots based on RNA-seq differential analysis were generated to visualize DEG distribution in four pairs of gastrointestinal cancers and adjacent noncancerous tissues, as shown in Figure 2A-D. Figure 2E showed that the expression levels of IGF2BP3 was significantly higher in esophageal cancer compared to adjacent noncancerous tissues(*P*<0.001). In addition, gastric cancer (Figure 2F), liver cancer (Figure 2G), and colorectal cancers (Figure 2H), have a similar expression profile. Compared to paired normal tissues, IGF2BP3 exhibited significant upregulation across all four types of gastrointestinal cancers (*P*<0.001), suggesting its potential involvement in tumorigenesis and progression. The mutational landscape of IGF2BP3 in gastrointestinal cancers was also explored, revealing that somatic alterations predominantly occur through amino acid substitutions, with missense mutations and single nucleotide polymorphisms (SNPs) being the most common types (Table 2).

### 3.4 Validation of IGF2BP3 expression in gastrointestinal cancers

**Figure 3:**
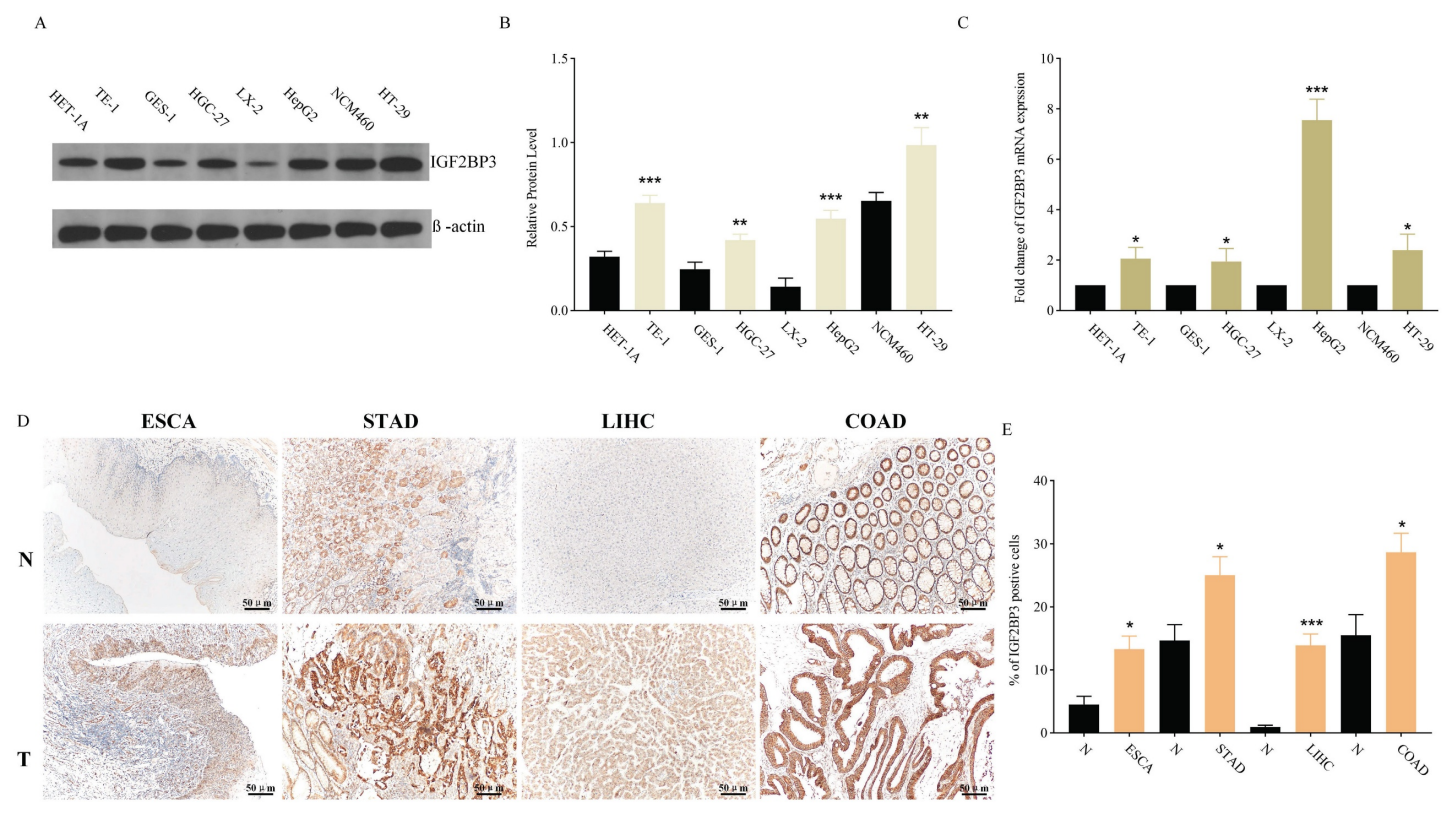
Validation of IGF2BP3 expression in gastrointestinal cancers. (A-B) WB for IGF2BP3 expression in HET-1A, TE-1, GES-1, HGC-27, LX-2, HepG2, NCM460, HT-29 cells. (C) QRT-PCR for IGF2BP3 expression in HET-1A, TE-1, GES-1, HGC-27, LX-2, HepG2, NCM460, HT-29 cells. (D-E) IGF2BP3 levels were determined by IHC staining in gastrointestinal cancers tissues.

In addition to bioinformatics analysis of IGF2BP3 expression in gastrointestinal cancers, experimental validations were conducted at both cellular and tissue levels. WB and QRT-PCR results showed that IGF2BP3 exhibited significantly higher expression at both protein and mRNA levels in all four tumor types compared to their corresponding normal tissues. The expression of IGF2BP3 was observed to be elevated in multiple cancer cell lines when compared to their normal counterparts: specifically, in TE-1 cells relative to HET-1A cells, in HGC-27 cells compared to GES-1 cells, in HepG2 cells versus LX-2 cells, and in HT-29 cells against NCM460 cells, with statistically significant differences noted (Figure 3A, B). Figure 3C demonstrated that the expression of IGF2BP3 mRNA was consistently elevated in gastrointestinal cancers cell lines. The upregulation of IGF2BP3 expression in tumor tissues across all four cancer types, as well as in their adjacent normal tissues, was further substantiated by IHC analysis (Figure 3D, E).

### 3.5 Related gene analysis of IGF2BP3 in gastrointestinal cancers

**Figure 4:**
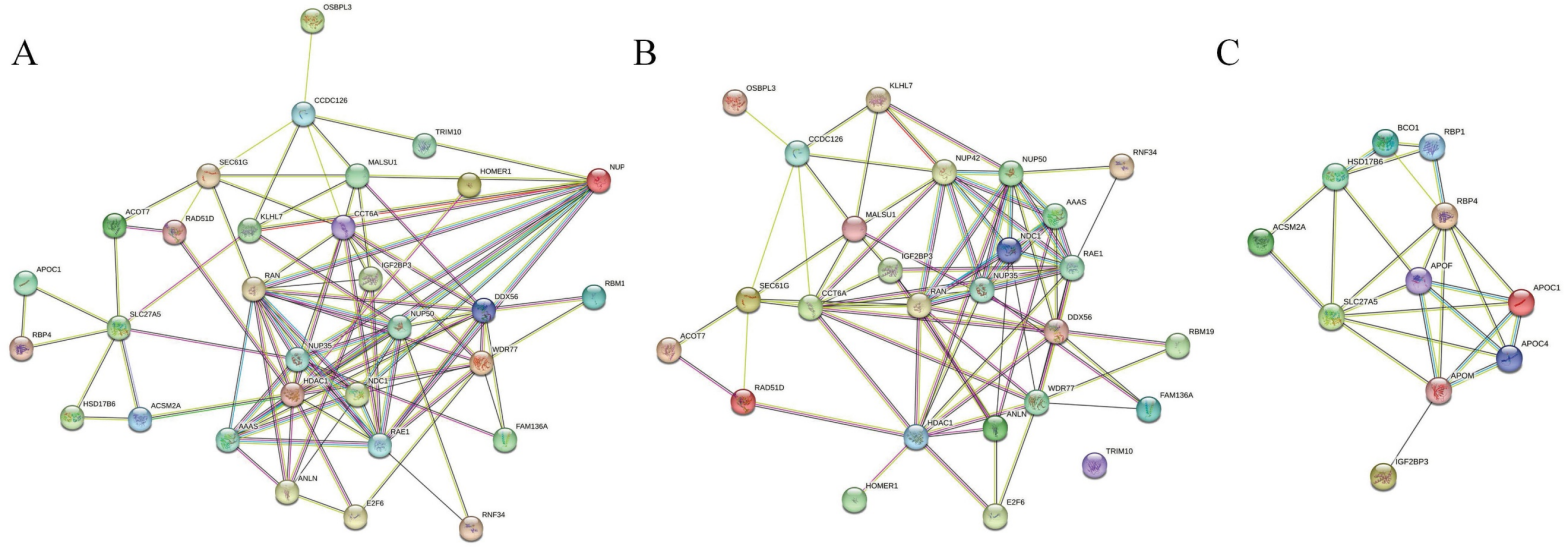
PPI network analysis of IGF2BP3 and its associated genes. (A) PPI network of genes correlated with IGF2BP3. (B) PPI network of genes positively correlated with IGF2BP3. (C) PPI network of genes negatively correlated with IGF2BP3.

We further investigated genes related to IGF2BP3 in four types of gastrointestinal cancers. In esophageal cancer, four genes were positively correlated with IGF2BP3: KLHL7, C7orf30, NUPL2, and RAD51L3. In colorectal cancer, six genes were positively correlated: ANLN, ACOT7, OSBPL3, SEC61G, DDX56, and TRIM10. In liver cancer, ten genes were positively correlated and five genes were negatively correlated with IGF2BP3: WDR77, RAN, CCT6A, HDAC1, FAM136A, RBM19, TMEM48, E2F6, RNF34, HOMER1, RBP4, APOC1, ACSM2A, HSD17B6, and SLC27A5. However, no related genes were found in gastric cancer (Table 3). Subsequently, PPI network analysis was performed on all relevant genes associated with IGF2BP3 (Figure 4A), all positively correlated genes (Figure 4B), and all negatively correlated genes (Figure 4C).

### 3.6 Correlation Between IGF2BP3 and Clinicopathological Characteristics of gastrointestinal cancers

**Figure 5:**
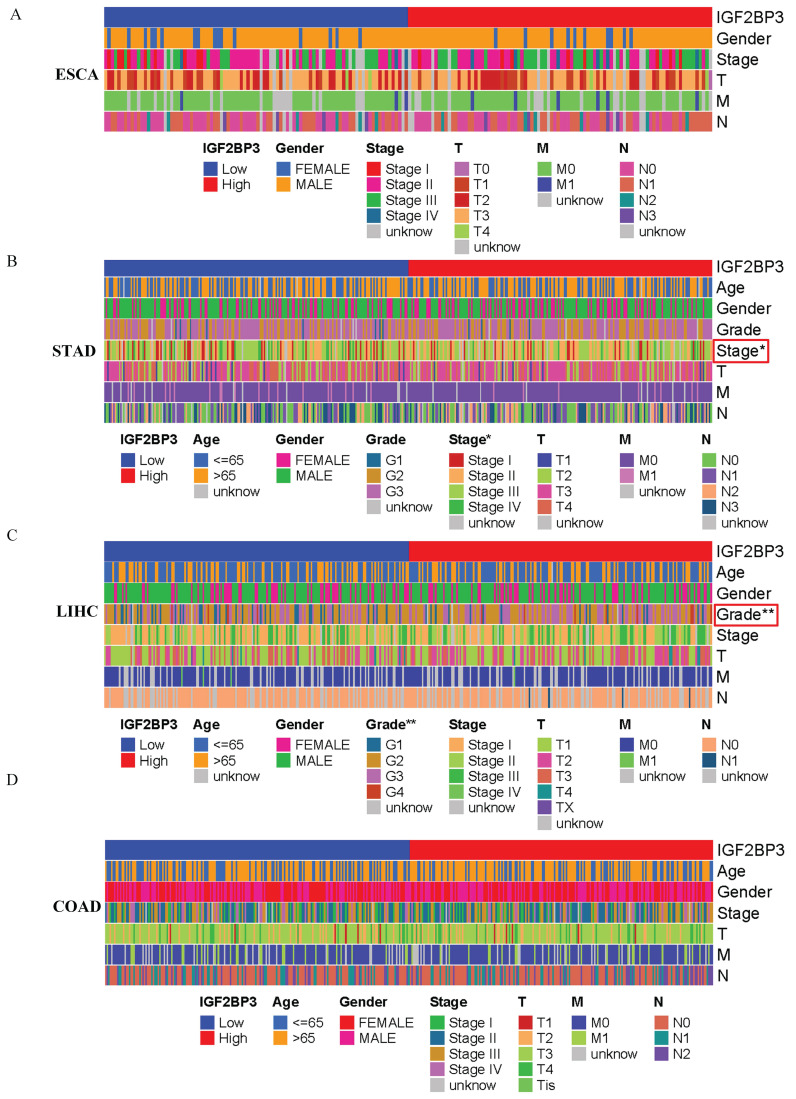
Correlation Between IGF2BP3 and Clinicopathological Characteristics of gastrointestinal cancers. (A-D) The relationship between IGF2BP3 expression and clinical characteristics of (A) esophageal, (B) gastric, (C) liver cancer, and (D) colorectal cancer.

Based on the median IGF2BP3 expression level from the TCGA database, patients were categorized into high-expression and low-expression groups. The correlation between IGF2BP3 expression and clinical features of patients with gastrointestinal tumors was then analyzed. Figure 5 shows that IGF2BP3 expression was significantly associated with cancer stage in gastric cancer and tumor grade in liver cancer (all *p*<0.05). However, there was no significant correlation between the level of IGF2BP3 expression and the sex, age, TMN grade of patients.

### 3.7 Diagnostic Efficacy of IGF2BP3 in gastrointestinal cancers

**Figure 6:**
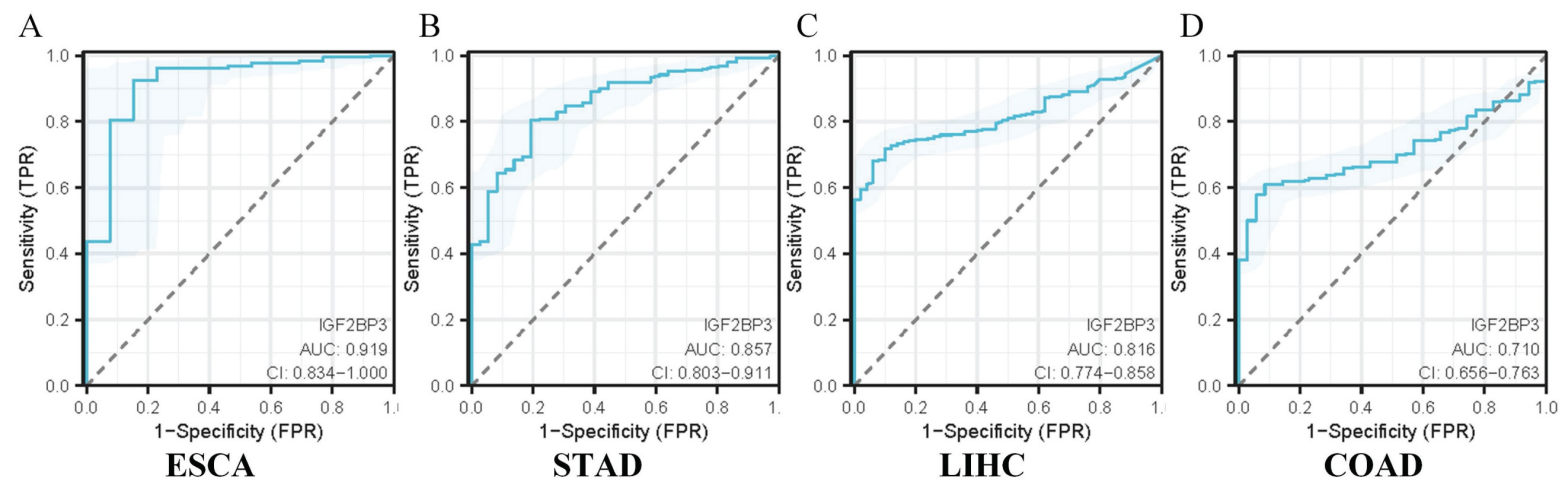
Diagnostic efficacy of IGF2BP3 in gastrointestinal cancers. (A) Diagnostic efficacy of IGF2BP3 in ESCA. (B) Diagnostic efficacy of IGF2BP3 in STAD. (C) Diagnostic efficacy of IGF2BP3 in LIHC. (D) Diagnostic efficacy of IGF2BP3 in COAD.

The diagnostic utility of IGF2BP3 in gastrointestinal cancers was assessed using ROC curve analysis. The confidence intervals for the impact of IGF2BP3 expression on diagnostic efficacy in gastrointestinal cancers were as follows: 0.834-1.000 for ESCA (Figure 6A), 0.803-0.911 for STAD (Figure 6B), 0.774-0.858 for LIHC (Figure 6C), and 0.656-0.763 for COAD (Figure 6D). The calculated AUC values were 0.919 (ESCA, Figure 6A), 0.857 (STAD, Figure 6B), 0.816 (LIHC, Figure 6C), and 0.710 (COAD, Figure 6D). According to established clinical criteria (AUC > 0.7: moderate diagnostic value; AUC>0.9: high diagnostic utility), IGF2BP3 demonstrated high diagnostic capacity for esophageal cancer, suggesting strong diagnostic value for its detection. It also showed moderate diagnostic capacity for the other three cancers.

### 3.8 Survival Analysis of IGF2BP3 in gastrointestinal cancers

**Figure 7:**
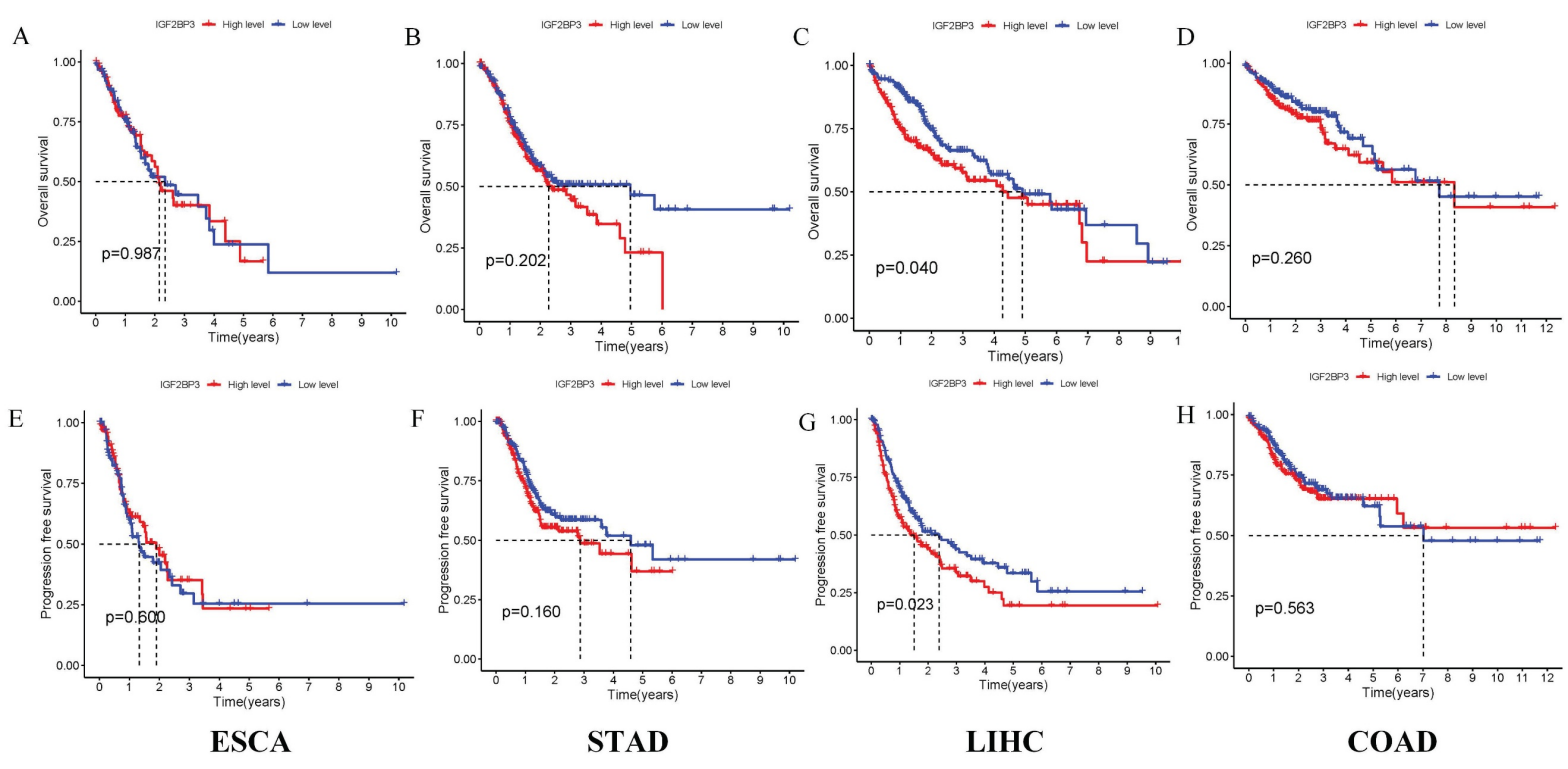
Prognostic Analysis of IGF2BP3 in gastrointestinal cancers. (A-D) Effect of IGF2BP3 expression on OS in gastrointestinal cancers patients. (E-H) Effect of IGF2BP3 expression on PFS in gastrointestinal cancers patients.

The correlation between IGF2BP3 expression and survival outcomes, including PFS, was assessed in four types of gastrointestinal cancers (Figure 7). Notably, high expression of IGF2BP3 significantly reduced survival time only in liver cancer, with this difference being statistically significant. In contrast, no significant survival differences were observed in the other three gastrointestinal cancers. These results suggest that IGF2BP3 may have particular relevance in liver cancer prognosis.

## 4. Discussion

Gastrointestinal cancers, encompassing those of the esophagus, stomach, liver, and colorectal regions, pose a considerable challenge to global health due to their widespread occurrence and high mortality rates. A comprehensive understanding of the molecular pathways that underpin the initiation and advancement of these malignancies is essential for enhancing early detection methods and treatment efficacy. IGF2BP3, belonging to the RNA-binding protein family, has emerged as a pivotal factor in regulating cellular growth and differentiation throughout development. Its potential as a diagnostic and prognostic marker for gastrointestinal tumors merits further investigation. Current research indicates that IGF2BP3 is implicated in the processes of proliferation, invasion, and resistance to therapeutic agents across a spectrum of tumor types 27. Furthermore, IGF2BP3 has been shown to interact with various signaling pathways, including the PI3K and MAPK pathways, which are critical for cancer cell survival and proliferation 28.

In this study, we primarily focused on the expression and mechanisms of IGF2BP3 in gastrointestinal cancers using bioinformatics methods. Initial analysis revealed that IGF2BP3 expression in gastrointestinal cancers exceeded the median expression levels observed across a broad range of cancers, laying the groundwork for further investigation into its diagnostic and prognostic significance in gastrointestinal cancers. This finding aligns with previous studies 29-32.

Using the TCGA database, volcano plots were generated to identify DEGs in gastrointestinal cancers. To further explore IGF2BP3 expression, box plots were created to compare its levels across four types of gastrointestinal cancers and their corresponding adjacent tissues. The analysis revealed significantly elevated expression of IGF2BP3 in all four cancer types. To experimentally validate these findings, WB, QRT-PCR, and IHC experiments were performed to assess IGF2BP3 expression at both the protein and RNA levels. These results were consistent with the findings from the box plots. Additionally, a literature review provided supporting data on the expression level of IGF2BP3 in gastrointestinal tumors 15, 16, 33-37.

To explore the molecular mechanisms associated with IGF2BP3 in gastrointestinal cancers, a thorough examination of gene mutations and genes related to IGF2BP3 was conducted. The most common type of mutation identified was the missense mutation, with SNPs representing the most frequently occurring variant. The analysis revealed the presence of IGF2BP3-related genes across three types of gastrointestinal cancers, excluding gastric cancer, indicating that mutations in IGF2BP3 could be crucial in the initiation and progression of these cancers. The results establish a basis for subsequent investigations into the molecular roles of IGF2BP3 within gastrointestinal cancers.

Additionally, the relationship between IGF2BP3 expression and clinical-pathological characteristics was examined. Elevated IGF2BP3 levels were significantly associated with advanced tumor stages in gastric cancer and higher tumor grades in colorectal cancer, indicating its involvement in tumor progression and differentiation. The diagnostic efficacy of IGF2BP3 was evaluated through ROC curves, which revealed that IGF2BP3 displayed the highest diagnostic accuracy for esophageal cancer and the lowest for colorectal cancer. Survival analysis using Kaplan-Meier curves derived from the TCGA database indicated that high IGF2BP3 expression was associated with reduced survival times only in liver cancer patients, while no significant differences were noted for the other three types of gastrointestinal cancers. These findings corroborate those of Sun et al. 38, which also found that high IGF2BP3 expression correlates with more aggressive tumor features and poorer outcomes, particularly in liver cancer, where elevated IGF2BP3 levels were linked to shortened OS and PFS. These results highlight the potential role of IGF2BP3 in the pathogenesis of liver cancer.

Despite the significant insights provided by this study, certain limitations exist. The role of IGF2BP3 in gastrointestinal cancers was primarily explored through bioinformatics and existing literature. Although this method holds merit, it is deficient in comprehensive molecular and cellular investigations to validate the functional role of IGF2BP3 in gastrointestinal cancers. Moreover, the lack of actual patient data from gastrointestinal cancers in this study constrains our capacity to reinforce and authenticate our conclusions. Future investigations will seek to overcome these constraints by integrating these experimental methodologies.

In summary, this study demonstrates that IGF2BP3 is highly expressed in gastrointestinal cancers and is associated with both diagnostic and prognostic factors in these cancers. These findings could provide valuable insights for clinical practice and offer potential avenues for future research.

## Figures and Tables

**Table 1:** Clinical parameters of patients

	ESCA	STAD	LIHC	COAD
N	184	403	349	404
Age	62	67	60.5	68.5
*P^a^*	0.145	0.270	0.560	0.113
Gender (%)				
Female	26(14.1)	143(35.5)	117(33.5)	198(49)
Male	158(85.9)	260(64.5)	232(66.5)	206(51)
*P^b^*	0.204	0.368	0.880	0.550
T (%)				
T1	31(16.8)	20(4.9)	167(47.8)	11(2.7)
T2	42(22.8)	84(20.8)	89(25.5)	70(17.3)
T3	88(47.8)	178(44.2)	78(22.4)	272(67.5)
T4	5(2.7)	113(28.1)	13(3.7)	50(12.3)
*P^c^*	0.859	0.359	0.314	0.830
N (%)				
N0	76(41.3)	120(29.7)	238(68.2)	234(58)
N1	69(37.5)	106(26.3)	4(1.1)	93(23)
N2	12(6.6)	77(19.1)	-	77(19)
N3	8(4.4)	81(20.1)	-	0
*P^d^*	0.926	0.602	0.134	0.670
M (%)				
M0	135(73.4)	358(88.9)	252(72.2)	291(72)
M1	9(4.9)	26(6.5)	4(1.2)	60(14.9)
*P^e^*	0.587	0.088	0.599	0.943
Grade (%)				
G1	-	12(3%)	49(14.1)	-
G2	-	141(35)	166(47.5)	-
G3	-	241(59.8)	118(33.8)	-
G4	-	-	12(3.4)	-
*P^f^*	-	0.561	0.024	-
Stage (%)				
Stage I	18(9.7)	54(13.4)	158(45.2)	69(17.1)
Stage II	78(42.4)	121(30.1)	81(23.2)	150(37.2)
Stage III	56(30.4)	166(41.2)	84(24.1)	114(28.3)
Stage IV	9(4.9)	38(9.4)	5(1.5)	60(14.9)
*P^g^*	0.745	0.046	0.548	0.980

Notes: The p-value represents the statistical difference of IGF2BP3 expression in different clinicopathological characteristics. The number of unclassified cases has not been showed. a: Age; b: Gender; c: T; d: N; e: M; f: Grade; g: Stage

**Table 2:** Gene mutations of IGF2BP3 in gastrointestinal cancers

Study of Origin	Sample ID	Protein Change	Mutation Type	Variant Type	Exon
LIHC	TCGA-CC-A7II-01	K36*	Nonsense	SNP	1
LIHC	TCGA-ED-A7PZ-01	L196M	Missense	SNP	6
ESCA	TCGA-L5-A893-01	S127A	Missense	SNP	5
STAD	TCGA-FP-A4BE-01	I302Lfs*17	FS del	DEL	8
STAD	TCGA-HU-A4GN-01	I302Nfs*2	FS ins	INS	8
STAD	TCGA-BR-8680-01	R551I	Missense	SNP	15
STAD	TCGA-VQ-A8P2-01	T407A	Missense	SNP	11
STAD	TCGA-CG-4437-01	I302N	Missense	SNP	8
STAD	TCGA-R5-A805-01	X402_splice	Splice	SNP	11
STAD	TCGA-D7-A6EY-01	X440_splice	Splice	SNP	12
STAD	TCGA-VQ-A8PT-01	P90H	Missense	SNP	3
COAD	TCGA-G4-6315-01	R169*	Nonsense	SNP	6
COAD	TCGA-AA-3977-01	E62*	Nonsense	SNP	2
COAD	TCGA-AA-A010-01	A240D	Missense	SNP	7
COAD	TCGA-AA-A024-01	Q224H	Missense	SNP	6
COAD	TCGA-AG-A002-01	K280N	Missense	SNP	8
COAD	TCGA-AG-A002-01	A237V	Missense	SNP	7
COAD	TCGA-AD-6964-01	P395L	Missense	SNP	10
COAD	TCGA-CM-6679-01	A160T	Missense	SNP	6
COAD	TCGA-D5-6929-01	K300N	Missense	SNP	8
COAD	TCGA-D5-6931-01	M453L	Missense	SNP	12

**Table 3:** Related genes analysis of IGF2BP3 in gastrointestinal cancers

Cancer Type	Related Gene	Correlation	Coefficient
ESCA	KLHL7	Positive	0.63
ESCA	C7orf30	Positive	0.62
ESCA	NUPL2	Positive	0.61
ESCA	RAD51L3	Positive	0.6
COAD	ANLN	Positive	0.44
COAD	ACOT7	Positive	0.39
COAD	OSBPL3	Positive	0.38
COAD	SEC61G	Positive	0.37
COAD	DDX56	Positive	0.37
COAD	TRIM10	Positive	0.37
LIHC	WDR77	Positive	0.62
LIHC	RAN	Positive	0.62
LIHC	CCT6A	Positive	0.61
LIHC	HDAC1	Positive	0.6
LIHC	FAM136A	Positive	0.6
LIHC	RBM19	Positive	0.6
LIHC	TMEM48	Positive	0.6
LIHC	E2F6	Positive	0.6
LIHC	RNF34	Positive	0.6
LIHC	HOMER1	Positive	0.6
LIHC	RBP4	Negative	-0.36
LIHC	APOC1	Negative	-0.34
LIHC	ACSM2A	Negative	-0.34
LIHC	HSD17B6	Negative	-0.34
LIHC	SLC27A5	Negative	-0.34
STAD	NA	NA	NA

## References

[1] Wang S, Zheng R, Li J, Zeng H, Li L, Chen R (2024). Global, regional, and national lifetime risks of developing and dying from gastrointestinal cancers in 185 countries: a population-based systematic analysis of GLOBOCAN. Lancet Gastroenterol Hepatol.

[2] Davila RE, Davila ML (2022). Recent Advancements in the Diagnosis and Treatment of Gastrointestinal Cancers. Gastroenterol Clin North Am.

[3] Huang J, Lucero-Prisno DE 3rd, Zhang L, Xu W, Wong SH, Ng SC (2023). Updated epidemiology of gastrointestinal cancers in East Asia. Nat Rev Gastroenterol Hepatol.

[4] Zhang Z, Zhou P, Liu M, Pei B (2023). Expression And Prognostic Role of PRDX1 In Gastrointestinal Cancers. J Cancer.

[5] Wen D, Xiao H, Gao Y, Zeng H, Deng J (2024). N6-methyladenosine-modified SENP1, identified by IGF2BP3, is a novel molecular marker in acute myeloid leukemia and aggravates progression by activating AKT signal via de-SUMOylating HDAC2. Mol Cancer.

[6] Zheng X, Li S, Yu J, Dai C, Yan S, Chen G (2023). N(6)-methyladenosine reader IGF2BP3 as a prognostic Biomarker contribute to malignant progression of glioma. Transl Cancer Res.

[7] Qin Z, Bai J, He H, Li B (2023). Expression and significance of m6A-RNA-methylation in oral cancer and precancerous lesion. Front Oncol.

[8] Wan W, Ao X, Chen Q, Yu Y, Ao L, Xing W (2022). METTL3/IGF2BP3 axis inhibits tumor immune surveillance by upregulating N(6)-methyladenosine modification of PD-L1 mRNA in breast cancer. Mol Cancer.

[9] Liu C, Dou X, Zhao Y, Zhang L, Zhang L, Dai Q (2024). IGF2BP3 promotes mRNA degradation through internal m(7)G modification. Nat Commun.

[10] Lu Y, Zhu J, Zhang Y, Li W, Xiong Y, Fan Y (2024). Lactylation-Driven IGF2BP3-Mediated Serine Metabolism Reprogramming and RNA m6A-Modification Promotes Lenvatinib Resistance in HCC. Adv Sci (Weinh).

[11] Xiang M, Liu L, Wu T, Wei B, Liu H (2023). RNA-binding proteins in degenerative joint diseases: A systematic review. Ageing Res Rev.

[12] Tao Y, Zhang Q, Wang H, Yang X, Mu H (2024). Alternative splicing and related RNA binding proteins in human health and disease. Signal Transduct Target Ther.

[13] Bertoldo JB, Müller S, Hüttelmaier S (2023). RNA-binding proteins in cancer drug discovery. Drug Discov Today.

[14] Xiong Q, Zhang Y (2023). Small RNA modifications: regulatory molecules and potential applications. J Hematol Oncol.

[15] Chen L, Hu B, Song X, Wang L, Ju M, Li Z (2021). m(6)A RNA Methylation Regulators Impact Prognosis and Tumor Microenvironment in Renal Papillary Cell Carcinoma. Front Oncol.

[16] Liu X, He H, Zhang F, Hu X, Bi F, Li K (2022). m6A methylated EphA2 and VEGFA through IGF2BP2/3 regulation promotes vasculogenic mimicry in colorectal cancer via PI3K/AKT and ERK1/2 signaling. Cell Death Dis.

[17] Li B, Zhao R, Qiu W, Pan Z, Zhao S, Qi Y (2022). The N(6)-methyladenosine-mediated lncRNA WEE2-AS1 promotes glioblastoma progression by stabilizing RPN2. Theranostics.

[18] Oh J, Hwa C, Jang D, Shin S, Lee SJ, Kim J (2022). Augmentation of the RNA m6A reader signature is associated with poor survival by enhancing cell proliferation and EMT across cancer types. Exp Mol Med.

[19] Gui Z, Li J, Li J, Li X, Chen L, Ma Z (2023). Berberine promotes IGF2BP3 ubiquitination by TRIM21 to induce G1/S phase arrest in colorectal cancer cells. Chem Biol Interact.

[20] Chen P, Xu J, Cui Z, Wu S, Xie T, Zhang X (2023). Multi-omics analysis of N6-methyladenosine reader IGF2BP3 as a promising biomarker in pan-cancer. Front Immunol.

[21] Chen LJ, Liu HY, Xiao ZY, Qiu T, Zhang D, Zhang LJ (2023). IGF2BP3 promotes the progression of colorectal cancer and mediates cetuximab resistance by stabilizing EGFR mRNA in an m(6)A-dependent manner. Cell Death Dis.

[22] Ge L, Rui Y, Wang C, Wu Y, Wang H, Wang J (2024). The RNA m(6)A reader IGF2BP3 regulates NFAT1/IRF1 axis-mediated anti-tumor activity in gastric cancer. Cell Death Dis.

[23] Su T, Zhang N, Wang T, Zeng J, Li W, Han L (2023). Super Enhancer-Regulated LncRNA LINC01089 Induces Alternative Splicing of DIAPH3 to Drive Hepatocellular Carcinoma Metastasis. Cancer Res.

[24] Huang J, Mao L, Lei Q, Guo AY (2024). Bioinformatics tools and resources for cancer and application. Chin Med J (Engl).

[25] Matsuoka T, Yashiro M (2024). Bioinformatics Analysis and Validation of Potential Markers Associated with Prediction and Prognosis of Gastric Cancer. Int J Mol Sci.

[26] Uesaka K, Oka H, Kato R, Kanie K, Kojima T, Tsugawa H (2022). Bioinformatics in bioscience and bioengineering: Recent advances, applications, and perspectives. J Biosci Bioeng.

[27] Wang C, Zhang M, Liu Y, Cui D, Gao L, Jiang Y (2023). CircRNF10 triggers a positive feedback loop to facilitate progression of glioblastoma via redeploying the ferroptosis defense in GSCs. J Exp Clin Cancer Res.

[28] Chen X, Zhu X, Shen X, Liu Y, Fu W, Wang B (2023). IGF2BP3 aggravates lung adenocarcinoma progression by modulation of PI3K/AKT signaling pathway. Immunopharmacol Immunotoxicol.

[29] Jin W, Yao Y, Fu Y, Lei X, Fu W, Lu Q (2024). WTAP/IGF2BP3-mediated GBE1 expression accelerates the proliferation and enhances stemness in pancreatic cancer cells via upregulating c-Myc. Cell Mol Biol Lett.

[30] Li K, Guo J, Ming Y, Chen S, Zhang T, Ma H (2023). A circular RNA activated by TGFβ promotes tumor metastasis through enhancing IGF2BP3-mediated PDPN mRNA stability. Nat Commun.

[31] Yang F, Ma Q, Huang B, Wang X, Pan X, Yu T (2023). CircNFATC3 promotes the proliferation of gastric cancer through binding to IGF2BP3 and restricting its ubiquitination to enhance CCND1 mRNA stability. J Transl Med.

[32] Imamura M, Wanibuchi S, Yamamoto Y, Kojima H, Ono A, Kasahara T (2021). Improving predictive capacity of the Amino acid Derivative Reactivity Assay test method for skin sensitization potential with an optimal molar concentration of test chemical solution. J Appl Toxicol.

[33] Cheng K, Liu S, Li C, Zhao Y, Wang Q (2023). IGF2BP3/HIF1A/YAP signaling plays a role in driving acute-on-chronic liver failure through activating hepatocyte reprogramming. Cell Signal.

[34] Tang J, Wang S, Weng M, Guo Q, Ren L, He Y (2023). The IGF2BP3-COPS7B Axis Facilitates mRNA Translation to Drive Colorectal Cancer Progression. Cancer Res.

[35] Guo Z, Zhang X, Lin C, Huang Y, Zhong Y, Guo H (2022). METTL3-IGF2BP3-axis mediates the proliferation and migration of pancreatic cancer by regulating spermine synthase m6A modification. Front Oncol.

[36] Huang GW, Chen QQ, Ma CC, Xie LH, Gu J (2021). linc01305 promotes metastasis and proliferation of esophageal squamous cell carcinoma through interacting with IGF2BP2 and IGF2BP3 to stabilize HTR3A mRNA. Int J Biochem Cell Biol.

[37] Ma Q, Yang F, Huang B, Pan X, Li W, Yu T (2022). CircARID1A binds to IGF2BP3 in gastric cancer and promotes cancer proliferation by forming a circARID1A-IGF2BP3-SLC7A5 RNA-protein ternary complex. J Exp Clin Cancer Res.

[38] Sun X, Ye G, Li J, Shou H, Bai G, Zhang J (2023). Parkin regulates IGF2BP3 through ubiquitination in the tumourigenesis of cervical cancer. Clin Transl Med.

